# Books

**Published:** 1889-01

**Authors:** 


A SYTEM OF GYNECOLOGY.—By American Authors, edited by Matthew D. Mann, A. M , M. D , Professor of Obstetrics and Gynecology, in the medical department of the University of Buffalo N. Y. Vol. second. Illustrated with four colored plates and three hundred and sixty-one engravings on wood.—Philadelphia, Lea Brothers & Co., 1888.

This, the second volume of this great work contains no less than 1180 large octavo pages, elegantly and substantially bound in leather, printed in large, plain and beautiful type, with admirable typographisal execution. The illustrations are beautiful, and the following subjects ably and elaborately treated by the Authors as mentioned.

*Diseases of the Vagina* by Charles Carroll Lee, A. M., M. D. of New York.
*Hystero Neuroses*, by George J. Englemen, M. D., of St. Louis, Mo.
*Extra Uterine Gestation*, by T. Gaillard Thomas, M. D., of New York.
*Tumors of the Breast*, by Samuel W. Gross, M. D., LL. D., of Philadelphia.
*Diseases of the Breast other than Tumors*, by Roswell Park, A. M., M D.
*Fistulae*, by Edward W. Jenks, M. D., LL. D.
*Diseases of the Bladder and Uterus*, by W. H. Baker, M. D.
*Non Malignant Tumors of the Uterus*, by R. Stansberry Sutton, A. M., M. D. LL. D.
*The Malignant Diseases of the Uterus*, by W. T. Lusk, M. D.
*Lacerations of the Cervix Uteri*, by Bache McEvers Emmet, M. D.
*Chronic inversion of the Uterus*, by Samuel C. Busey, M. D., L. L. D.
*Injuries and Lacerations of the Perineum and pelvic floor*, by Howard A. Kelly, M. D.
*The treatment of ovarian and of extra-ovarian tumors*, by Wm. Coodell, A. M., M. D.
*Diseases of the Ovaries*, by Robt. Battey, M. D. and Henry C. Coe, A. M., M. D.
*Diseases of Fallopean Tubes*, by Henry C. Coe, A. M., M. D. and W. Gill Wylie, M. D.
*The pathology of ovarian tumors*, by Stephen W. Howell, A. M., M. D.
*The Clinical History and Diagnosis of Pelvic tumors, other than uterine and tubal*, by Mathew D. Mann, A. M., M. D.
*Displacements of the Uterus*, by George T. Harrison, M. D.

